# PRELIMINARY EFFECTIVENESS AND PRODUCTION TIME AND COSTS OF THREE-DIMENSIONAL PRINTED ORTHOSES IN CHRONIC HAND CONDITIONS: AN INTERVENTIONAL FEASIBILITY STUDY

**DOI:** 10.2340/jrm.v56.39946

**Published:** 2024-05-14

**Authors:** Tanja OUD, Johannes A. BOGAARDS, Frans NOLLET, Merel-Anne BREHM

**Affiliations:** 1 Amsterdam UMC, University of Amsterdam, Department of Rehabilitation Medicine, Meibergdreef 9, Amsterdam; 2 Amsterdam Movement Sciences, Rehabilitation & Development, Amsterdam; 3 Amsterdam UMC, University of Amsterdam, Department of Epidemiology & Data Science, Amsterdam; 4 Amsterdam Public Health, Methodology, Amsterdam, the Netherlands

**Keywords:** feasibility study, hand, orthotic devices, patient reported outcome measures, printing, three-dimensional

## Abstract

**Objective:**

To assess the preliminary effectiveness of three-dimensional printed orthoses compared with conventionally custom-fabricated orthoses in persons with chronic hand conditions on performance of daily activities, hand function, quality of life, satisfaction, and production time and costs.

**Design:**

Interventional feasibility study.

**Subjects:**

Chronic hand orthotic users (*n* = 21).

**Methods:**

Participants received a new three-dimensional printed orthosis according to the same type as their current orthosis, which served as the control condition. Primary outcome was performance of daily activities (Patient-Reported Outcomes Measurement Information System–Upper Extremity; Michigan Hand Questionnaire). Secondary outcomes were hand function, quality of life, and satisfaction. Furthermore, production time and costs were recorded.

**Results:**

At 4 months’ follow-up, no significant differences were found between three-dimensional printed orthoses and participants’ existing conventional orthoses on activity performance, hand function, and quality of life. Satisfaction with the three-dimensional printed orthosis was significantly higher and the production time and costs for three-dimensional printed orthoses were significantly lower compared with conventional orthoses. The three-dimensional printed orthosis was preferred by 79% of the participants.

**Conclusions:**

This feasibility study in chronic hand conditions suggests that three-dimensional printed orthoses are similar to conventional orthoses in terms of activity performance, hand function, and quality of life. Satisfaction, and production time and costs favoured the three-dimensional printed hand orthoses.

LAY ABSTRACT

The aim of this study was to compare performance of daily activities when using a newly made three-dimensional printed hand orthosis with existing custom-made conventional orthoses in persons with chronic hand conditions. We also compared hand function, orthosis satisfaction, quality of life, and production time and costs. Twenty-one participants were included in the study, and 19 of them were provided with a new three-dimensional printed orthosis in accordance with the same type as their existing orthosis. After four months of wearing the new three-dimensional printed orthosis, no differences were found on activity performance, hand function, and quality of life compared with the existing conventional orthosis. Satisfaction with the three-dimensional printed orthosis was higher compared with the conventional orthosis, and of the 19 participants, 15 (79%) preferred the three-dimensional printed hand orthosis. The production time and costs of the three-dimensional printed orthoses were more than halved compared with conventional orthoses.

Individuals with chronic hand conditions are often prescribed hand orthoses to reduce impairments (e.g. pain, loss of grip strength, spasticity, and joint and/or muscle contractures) (1, 2), which in turn helps to improve the performance of activities of daily living (ADL) (3–5). Because hand orthoses in chronic conditions are commonly worn on a daily basis, they need to fit well and be durable. Therefore, orthoses for chronic hand conditions are usually custom-fabricated, which is labour intensive and time-consuming (1).

In the past decade, three-dimensional (3D) printing has emerged in the field of orthotics as a promising and less labour-intensive alternative to manufacture hand orthoses (6–10). In a recent case series on chronic hand conditions, we found that the production time of 3D-printed orthoses was reduced to half the duration of producing conventional, custom-fabricated orthoses. Furthermore, participants experienced 3D scanning for orthosis fitting as more comfortable than conventional casting (9). Previous studies have also demonstrated that satisfaction with 3D-printed orthoses was similar or higher compared with the investigated control orthoses (9–11).

Despite these advantages of 3D printing, to date there is little evidence on the effectiveness of 3D-printed hand orthoses compared with conventional custom-fabricated hand orthoses in chronic conditions (6, 7). In particular, there is a lack of evidence on ADL performance, although this is one of the most important goals for which a hand orthosis is prescribed. Currently, only 2 studies have investigated the effects of 3D-printed hand orthoses on ADL performance in chronic hand conditions (10, 12). However, these studies compared 3D-printed orthoses with plaster casts (10) and prefabricated orthoses (12), and not with durable custom-fabricated hand orthoses, which have different characteristics. Therefore, the results of these 2 studies are not generalizable to custom-fabricated orthoses. Additionally, information on the costs of manufacturing 3D-printed orthoses is lacking (6), which is an important aspect considering the rising healthcare costs, as illustrated by a 27% rise in the total costs for upper extremity orthoses in the Netherlands from 2018 to 2022 (13).

To address these knowledge gaps, we set up an interventional feasibility study following Bowen’s Feasibility Framework (14). This framework includes 8 focus areas: acceptability, demand, implementation, practicality, adaptation, integration, expansion, and limited efficacy testing. For our study, we addressed the areas of limited efficacy testing, acceptability, and practicality. Limited efficacy testing focuses on whether the intervention shows promise of being successful with the intended population, while acceptability refers to the extent to which a new intervention is judged as suitable, satisfying, or attractive to both the intervention recipients and deliverers. Finally, practicality relates to the extent to which an intervention can be delivered with regard to time, resources, and commitment (14). Based on these 3 focus areas the study aims were to (*i*) assess the preliminary effectiveness of 3D-printed hand orthoses compared with conventional custom-fabricated hand orthoses on ADL performance, general hand function, and quality of life in persons with chronic hand conditions (limited efficacy testing); (*ii*) evaluate satisfaction with the orthosis and the experiences of participants and orthotists with the 3D manufacturing process (acceptability); and (*iii*) assess the production time and costs of both orthoses (practicality). Insights into the preliminary effectiveness on ADL performance, satisfaction, and costs of 3D-printed hand orthoses compared with conventional custom-fabricated orthoses can be used as input for a randomized comparison in future cost-effectiveness studies.

## METHODS

### Study design

A prospective, interventional feasibility study was performed, using a within-subject design, with the conventional orthoses worn by the participants at baseline serving as the control condition. Assessments were done 2 weeks prior to the intervention (T1) and at baseline (T2) for the conventional orthosis, and at 1 month (T3) and 4 months (T4) after provision of the newly made 3D-printed orthosis. The study was reported according to the guidelines of the Consolidated Standards of Reporting Trials (CONSORT) 2010 statement; extension to randomized pilot and feasibility trials (15).

### Study population

Participants were recruited from the databases of 3 branches of the orthotic company OIM Orthopedie (Zwolle, Noordwijkerhout and Breda, The Netherlands). They were enrolled between April 2022 and mid-June 2022. Eligible participants were those who: (*i*) had a stable, chronic hand condition due to a neuromuscular disease, neurological disorder, musculoskeletal disorder, or injury; (*ii*) were aged ≥ 18 years; and (*iii*) were wearing a conventional custom-fabricated circular thumb orthosis (TO), wrist orthosis (WO), or wrist–thumb orthosis (WTO) during ADL for at least 2 years (to be eligible for reimbursement of the orthosis via the Health Insurance). Persons who were already wearing a 3D-printed orthosis, or who wore a silver splint (not manufactured via the conventional method by an orthotist), a broken orthosis (no valid comparator), an orthosis for a dysfunctional hand, or a night orthosis (in both cases the orthosis is not used during ADL) were excluded. Individuals who did not understand the explanations and instructions in Dutch were also excluded.

### Study procedures

Participants visited the department of Rehabilitation Medicine of Amsterdam UMC, location AMC, once to sign informed consent, and, subsequently, a certified hand therapist checked the inclusion and exclusion criteria. After study enrolment, demographics (e.g. age, gender), clinical characteristics (e.g. cause of chronic hand condition, goals of orthosis use, frequency of orthosis use), and type and material of the existing conventional orthoses were inventoried. In addition, outcomes with the participants’ conventional orthosis were assessed (T1) and reassessed 2 weeks later (T2). Subsequently, the 3D-printed hand orthosis intervention started, which was provided at one of the 3 branches of OIM Orthopedie. One week after delivering the 3D-printed orthosis, the investigator contacted the participants by phone to check the fit and identify any issues (e.g. pressure points, skin irritation, and inadequate immobilization/stiffness) of the orthosis. If the fit of the 3D-printed orthosis was not adequate, the orthosis was revised. Otherwise, follow-up assessments were planned after 1 month (T3) and 4 months (T4) of using the orthosis. For the assessments, questionnaires were sent digitally using an electronic data capture system (Castor EDC, Amsterdam, The Netherlands). A schematic overview of the study design is included in our previously published protocol article (16).

*Intervention.* Each 3D-printed orthosis was made according to the same type as the participant’s current conventional orthosis (i.e. a circular TO, WO, or WTO). The participants’ affected hand and forearm were scanned while they held their hand and fingers in the correct position. The scan was made by an experienced orthotist using a calibrated iPad and accessory app (Spentys, Vorst, Belgium). If needed, the scan was post-processed for areas requiring pressure relief. Based on the 3D-scan and standardized model scripts, the orthosis was digitally designed by Spentys. The orthoses were printed in thermoplastic polyurethane and made water resistant by vapor smoothing (ZiggZagg, Aalter, Belgium). Fitting and alignment of the orthosis were checked before delivery. Local adjustments were made if required or, if necessary, a new scan was made to design and print a new orthosis. To ensure that the 3D intervention was performed uniformly across the 3 OIM branches, the orthotists were trained in positioning the forearm and hand, use of the iPad for scanning, and use of the Spentys app for ordering the orthosis.

*Control condition.* Participants’ existing conventional orthoses included circular TO, WO, and WTO made of resin, thermolyn supra flexible, leather, or silicone. The conventional orthoses were designed manually based on a plaster cast of the participant’s affected hand and forearm, as previously described (9). Fitting and alignment were checked prior to delivery, and adjustments were made to the orthosis if required.

### Outcomes

*Primary outcome.* The primary outcome, ADL performance, was assessed with the validated Dutch-Flemish Patient Reported Outcomes Measurement Information System – Upper Extremity (DF-PROMIS-UE) (17, 18) and with the ADL domain of the valid and reliable Michigan Hand Questionnaire – Dutch language version (MHQ-DLV) (19–21). For the DF-PROMIS-UE, a custom short form (25 items) was created from the original 46-item bank. For each item, participants rated whether they were able to perform that activity (score range: 4 or 5 = without any difficulties to 1 = unable to do) (17). The total score is expressed as a T-score, which is a standardized score, ranging from 11.5 to 57.4 for the 25-item short form. For each activity of the DF-PROMIS-UE, a question was added asking whether the participants used their orthosis for that specific activity. With the MHQ-DLV ADL domain, 17 activities were rated on a 5-point scale (range: 1 = not difficult at all to 5 = very difficult). The converted sum score ranges from 0–100. For both outcome measures, higher scores indicate better ADL performance.

*Secondary outcomes.* Secondary outcomes included (*i*) general hand function (assessed with MHQ-DLV domains overall hand function, work performance, pain, hand aesthetics, and hand satisfaction), (*ii*) quality of life (assessed with the EuroQoL 5-Dimension 5-Level (EQ-5D-5L; domains mobility, self-care, usual activities, pain/discomfort, and anxiety/depression) and the EQ visual analogue scale [VAS]), and (*iii*) orthosis satisfaction (Client Satisfaction with Device [CSD] module and Dutch version of the Quebec User Evaluation of Satisfaction with Assistive Technology [D-QUEST]). Further, production time and costs were measured for both orthoses, as well as the experiences of participants and orthotists with the 3D-printing intervention. Descriptions of the secondary outcomes have been provided in the protocol article (16). A more detailed description of the assessment of orthosis satisfaction and production time and costs is given below.

Orthosis satisfaction was assessed with the CSD, which we translated and cross-culturally adapted into Dutch (D-CSD). The D-CSD was found to be valid and reliable for use in our population (22). Ten items were rated on a 5-point Likert scale (0 = strongly disagree to 4 = strongly agree), resulting in a sum score of 0–40. In addition, the device subscale of the valid and reliable D-QUEST was used (23–25), scoring 8 characteristics of the orthosis on a 5-point scale (1 = not satisfied at all to 5 = very satisfied; sum score of 8–40).

The conventional orthoses worn by the participants were manufactured prior to the study and therefore the production time was not recorded. To be able to measure production time, prototype copies of circular conventional TOs, WOs, and WTOs of each included material were newly fabricated, and labour time for each manufacturing step was recorded. To fairly compare labour time of the 3D-printed orthoses with the conventional orthoses, only labour time for the first 3D-printed orthosis was taken in the analysis. Costs of the conventional and 3D-printed orthosis were calculated by summing the costs of the labour time needed to manufacture each orthosis, plus the material costs. In addition, we counted the number of visits for the 3D-printed orthoses. The visits for the conventional orthoses were counted from the records of OIM Orthopedie.

*Adverse events.* Adverse events that occurred with the 3D-printed orthosis were noted during the entire follow-up period.

### Sample size

This feasibility study was not powered to determine the effectiveness of 3D-printed orthoses. We considered 20 participants a sufficiently large sample to evaluate the preliminary effectiveness and to get an impression of the effect sizes and variances of the outcomes included.

### Statistical analysis

Demographics, clinical characteristics, orthosis properties, and primary and secondary outcomes were summarized with descriptive statistics.

We analysed the primary outcome (ADL performance) and secondary outcomes (general hand function, orthosis satisfaction, and quality of life [EQ-VAS]) with linear mixed models. The model incorporated random effects for each scheduled assessment on the patient level. The assumption on normality of the residuals was visually checked. The distribution of scores across EQ-5D-5L domains and the use of the orthosis during performance of the DF-PROMIS-UE activities at each time point were analysed with Fisher’s exact tests.

Additionally, a Hedges’ g was estimated for the DF-PROMIS-UE and MHQ-DLV ADL domain, as well as for the D-CSD and D-QUEST, to determine their responsiveness.

Contrary to the study protocol (16), we did not pool the results of the conventional orthosis obtained 2 weeks prior to the intervention (T1) and at baseline (T2), as it appeared that there was an anticipation effect in 2 out of 3 outcome measures (DF-PROMIS-UE: mean difference T2 vs T1: –1.6, *p* = 0.007; D-CSD: mean difference T2 vs T1: –2.1, *p* = 0.04). Therefore, we compared baseline (T2) data with those at 1 month (T3) and 4 months’ (T4) follow-up. For the D-QUEST and EQ-5D-5L, only T1 data were available and compared with T3 and T4. Differences in production time and costs between the 3D-printed and conventional orthoses were analysed with Wilcoxon signed-rank tests.

In all analyses, statistical uncertainty was expressed by means of 95% confidence intervals. Statistical significance was set at *p* < 0.05. We performed all analyses in R statistics, version 4.2.1 using packages mosaic, nlme, ggplot2, and effsize (R Foundation for Statistical Computing, Vienna, Austria).

## RESULTS

**Fig. 1:**
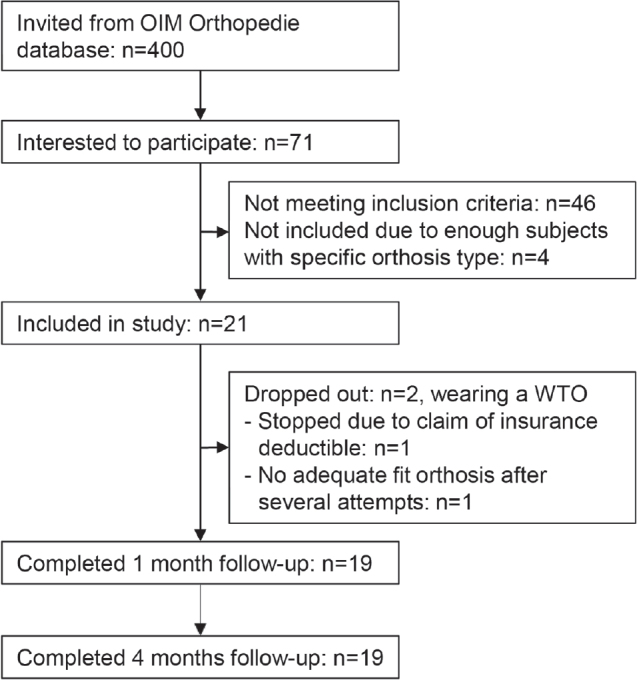
Participant flowchart.

We invited 400 individuals, of whom 71 were interested in participating. Based on the inclusion and exclusion criteria, 21 people could be included. Main reasons for ineligibility were not having the correct type of orthosis (*n* = 15) and not wearing the orthosis for > 2 years (n = 12). The demographic and clinical characteristics of the participants are presented in Table I. Nineteen participants completed the follow-up assessments. A flowchart shows how participants progressed through the study, including reasons for dropout (Fig. 1).

**Table I:** Demographic and clinical characteristics of participants at baseline

Factor	Participants (*n*=21)
Age, years, median (IQR)	63 (56–66)
Gender; male/female, n (%)	5 (24)/16 (76)
Cause chronic hand condition, *n* (%)	
Injury	1 (5)
Musculoskeletal disorder	16 (76)
Neuromuscular disorder	1 (5)
Neurological disorder	3 (14)
Orthosis; unilateral/bilateral, *n* (%)	8 (38) / 13 (62)
Type of orthosis*, *n* (%)	
Thumb orthosis	5 (24)
Wrist orthosis	8 (38)
Wrist–thumb orthosis	8 (38)
Orthosis material*, *n* (%)	
Silicone	12 (57)
Leather	5 (24)
Thermolyn supra flexible	4 (19)
Wearing days per week*, *n* (%)	
6–7 days	9 (43)
4–5 days	6 (29)
2–3 days	6 (29)
1 day	0 (0)
Wearing time per day*, *n* (%)	
24 hours	0 (0)
During daytime	6 (29)
During strenuous activities	15 (71)
Goals of use*, *n* (%)	
Improve ability to perform activities	15 (71)
Support the wrist and/or thumb	16 (76)
Support the wrist and/or thumb when performing activities	17 (81)
Reduce pain in wrist and/or thumb	19 (90)
Reduce pain in wrist and/or thumb when performing activities	8 (38)
Reduce fatigue in wrist and/or thumb	10 (48)

* Based on the type of conventional orthosis for which a 3D-printed orthosis was fabricated.

### Outcomes

**Fig. 2:**
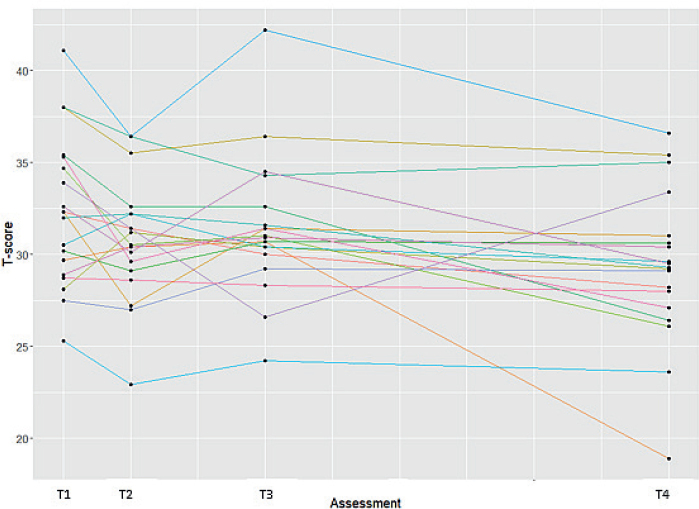
Individual effects on ADL performance as measured with the DF-PROMIS-UE. Assessments with conventional orthosis: T1=2 weeks prior to intervention, T2=baseline (i.e. 0 weeks); assessments with 3D-printed orthoses: T3=1 month after orthosis delivery, T4=4 months after orthosis delivery.

*Limited efficacy testing.* On group level, there was no significant difference in ADL performance on the DF-PROMIS-UE and MHQ-DLV ADL domain for 3D-printed orthoses compared with conventional orthoses at both 1 month and 4 months’ follow-up (Table II). There was a significant decrease in ADL performance on the DF-PROMIS-UE for the 3D-printed orthosis between 1 month and 4 months of follow-up (mean difference -2.07; 95% CI –3.76; –0.38). On an individual level, heterogeneity in effects was observed (Fig. 2). The effect sizes of ADL performance were small and somewhat higher for the DF-PROMIS-UE than for the MHQ-DLV ADL domain (Table III). With regard to the use of the orthosis during performance of the activities included in the DF-PROMIS-UE, no significant difference was found between the 3D-printed and conventional hand orthoses. The orthoses were most often used during the activities “carry a shopping bag or briefcase” (80%), “turning key” (70%), and “push door open after turning knob” (64%). The activities in which the orthosis was least worn were “drying back with towel” (17%) and “shampoo hair” (3%).

**Table II:** Effects on group level (*n*=19) of 3D-printed orthoses compared with conventional orthoses on the primary and secondary outcomes

		Conventional orthosis		3D-printed orthosis		Change over time		
		T1 Mean (SD) Median [range]	T2 Mean (SD) Median [range]	T3 Mean (SD) Median [range]	T4 Mean (SD) Median [range]	T3 vs T2 Mean difference [95% CI]	T4 vs T2 Mean difference [95% CI]	T4 vs T3 Mean difference [95% CI]
Primary outcome								
ADL performance	DF-PROMIS-UE (T-score)	32.3 (4.07) 32.3 [25.3–41.1]	30.8 (3.27) 30.5 [22.9–36.4]	31.4 (3.77) 30.9 [24.2–42.2]	29.3 (4.17) 29.3 [18.9– 36.6]	0.62 [–0.50; 1.73]	–1.46 [–3.01; 0.09]	–2.07 [–3.76; –3.38]
	MHQ-DLV ADL domain (sum score 0–100)	61.6 (14.3) 60.4 [41.1–88.6]	60.7 (16.6) 62.9 [26.8–95.7]	61.6 (18.0) 58.9 [27.1–97.5]	55.7 (21.4) [5.36–88.6]	0.85 [–4.14; 5.83]	–4.99 [–12.4; 2.38]	–5.84 [–13.8; 2.09]
Secondary outcomes								
General hand function	MHQ-DLV (sum score 0–100) Hand function Work Pain Hand aesthetics Hand satisfaction	50.8 (17.5) 47.5 [25.0–95.0] 45.3 (21.8) 40.0 [20.0–95.0] 42.1 (17.7) 40.0 [15.0–90.0] 67.9 (22.7) 68.8 [37.5–100] 41.0 (21.3) 37.5 [8.33–95.8]	47.9 (14.5) 45.0 [15.0–85.0] 45.0 (18.4) 45.0 [25.0–80.0] 45.5 (19.9) 40.0 [10.0–95.0] 71.2 (20.3) 75.0 [40.6–100] 37.4 (22.1) 35.4 [0.00–100]	51.6 (16.3) 50.0 [15.0–95.0] 48.7 (22.7) 50.0 [10.0–100] 49.7 (17.9) 50.0 [15.0–100] 74.8 (22.5) 81.3 [31.3–100] 44.7 (22.2) 41.7 [8.33–95.8]	48.4 (15.3) 50.0 [10.0–75.0] 44.2 (20.9) 50.0 [0.00–75.0] 47.4 (17.9) 45.0 [20.0–85.0] 70.4 (24.5) 75.0 [18.8–100] 45.0 (20.2) 43.8 [8.33–100]	3.68 [–1.19; 8.56] 3.68 [–5.49; 12.9] 4.21 [–1.41; 9.83] 3.62 [–4.87;12.1] 7.35 [0.36; 14.3]	0.53 [–5.53; 6.59] –0.79 [–9.73; 8.15] 1.84 [–6.61; 10.3] –0.82 [–10.0;8.38] 7.57 [–0.55; 15.7]	–3.16 [–9.30; 2.99] –4.47 [–13.2; 4.23] –2.37 [–10.1; 5.40] –4.44 [–10.1;1.17] 0.22 [–6.82; 7.26]
Quality of life	EQ-5D-5L VAS (sum score 0–100)	64.3 (25.0) 70.0 [5.00–95.0]	XXX^a^	69.3 (14.6) 74.0 [40.0– 97.0]	63.5 (20.6) 68.0 [3.00–95.0]	5.00 [–2.93; 12.9]^b^	–0.79 [–9.01; 7.43]^b^	–5.79 [–11.9; 0.27]
Orthosis satisfaction	D-CSD (sum score 0–40)	26.2 (6.39) 24.0 [18.0– 40.0]	24.1 (5.61) 24.0 [12.0–36.0]	28.2 (5.72) 28.9 [13.0–36.0]	29.2 (5.94) 30.0 [15.0–39.0]	4.04 [–0.02; 8.10]	5.11 [0.62; 9.59]	1.06 [–0.13; 2.26]
	D-QUEST (sum score 8–40)	30.5 (3.86) 31.0 [23.0–39.0]	XXX^a^	31.8 (4.35) 32.0 [22.0–40.0]	31.5 (5.47) 33.0 [20.0–39.0]	1.37 [–1.42; 4.15]^b^	1.05 [–2.25; 4.35]^b^	–0.32 [–1.79; 1.16]

3D: 3 dimensional; ADL: activities of daily living; D-CSD: Dutch Client Satisfaction with Device; DF-PROMIS-UE: Dutch-Flemish Patient-Reported Outcomes Measurement Information System – Upper Extremity; D-QUEST: Dutch version of the Quebec User Evaluation of Satisfaction with Assistive Technology; EQ-5D-5L: EuroQoL 5-Dimensions 5-Levels; MHQ-DLV: Michigan Hand Questionnaire Dutch language version; SD: standard deviation.

a For this questionnaire only T1 was performed for the conventional orthosis;

b T1 instead of T2.

**Table III:** Effect sizes for ADL performance and orthosis satisfaction

	T3 vs T2 Hedges’ g [95% CI]	T4 vs T2 Hedges’ g [95% CI]		T4 vs T3 Hedges’ g [95% CI]	
ADL performance					
DF-PROMIS-UE	0.17 [–0.47; 0.82] Negligible		–0.38 [–1.03; 0.27] Small		–0.51 [–1.17; 0.14] Medium
MHQ-DLV ADL domain	0.05 [–0.60; 0.69] Negligible		–0.26 [–0.90; 0.39] Small		–0.29 [–0.94; 0.36] Small
Orthosis Satisfaction					
D-CSD	0.32 [–0.33; 0.97]^a^ Small		0.48 [–0.17; 1.14]^a^ Small		0.18 [–0.47; 0.82] Negligible
D-QUEST	0.33 [–0.32; 0.97]^a^ Small		0.22 [–0.43; 0.86]^a^ Small		–0.06 [–0.71; 0.58] Negligible

Effect sizes are presented as Hedges’ g. Abbreviations: ADL: activities of daily living; DF-PROMIS-UE: Dutch-Flemish Patient-Reported Outcomes Measurement Information System – Upper Extremity; D-CSD: Dutch Client Satisfaction with Device; D-QUEST: Dutch version of the Quebec User Evaluation of Satisfaction with Assistive Technology; MHQ-DLV: Michigan Hand Questionnaire Dutch language version.

a T1 instead of T2.

Regarding the secondary outcomes, no differences were found for quality of life (separate domains and EQ VAS score) and the MHQ-DLV domains hand function, work, pain, and aesthetics at 1 and 4 months’ follow-up (Table II), except for the MHQ-DLV hand satisfaction domain, which had a significantly higher score at 1 month follow-up compared with baseline (mean difference: 7.35; 95% CI 0.36; 14.33).

Table II also shows a trend of (slight) improvement in ADL performance, general hand function, and quality of life at 1 month follow-up compared with baseline. A small decrease was seen at 4 months’ follow-up compared with 1 month follow-up (except for hand satisfaction).

*Acceptability.* At 1 and 4 months’ follow-up, orthosis satisfaction, as measured with the D-CSD, was higher for the 3D-printed orthosis compared with the conventional orthosis, with a significant difference at 1 month follow-up (mean difference: 5.11 95% CI: 0.62; 9.59, Table II). On the D-QUEST, there was a slight, non-significant higher orthosis satisfaction for the 3D-printed orthoses compared with conventional orthoses. The effect sizes were negligible to small, although the effect size of the D-CSD was slightly higher than that of the D-QUEST (Table III).

Of the 19 participants, 15 (79%) preferred the 3D-printed orthosis, with more precise fit, lower weight, and less transpiration being the most important reasons. Indicated points for improvement were the possibility to choose a different colour and the use of softer material. Four participants (leather orthosis, *n* = 2; silicone orthosis, *n* = 2) preferred the conventional orthosis, which they found to be more supportive and softer.

Regarding the treatment process, participants were mostly satisfied with the procedure of scanning, fitting, and delivery of the 3D-printed orthosis and the time required for the intervention (range: neutral to highly satisfied). Reported pros were that the 3D intervention takes less time (*n* = 13) and is more precise (*n* = 4). Mentioned cons were fewer possibilities for adjustments (*n* = 1) and the need to hold the scan position for too long (*n* = 1). The 3 orthotists were mostly neutral or satisfied with the treatment process (range: not satisfied at all [*n* = 2] to highly satisfied [*n* = 1]). The orthotists reported the same pros as the participants. In some cases, the orthotists had difficulty positioning the hand to get the correct scan. Other mentioned cons were the difficulty of adjusting the 3D-printed orthoses when needed and a relatively long delivery time of the 3D-printed orthosis.

*Practicality.* The production time and costs were significantly lower (*p* < 0.001) for 3D-printed orthoses (production time: median 129 min, IQR: 109–148 min, costs: median €187, IQR: €143–206) compared with the conventional orthoses (production time: median 269 min, IQR: 241–311 min; costs: median €398, IQR: €380–436). The mean number of visits was 3.2 and 2.5 for the 3D-printed (*n* = 19) and conventional orthoses (*n* = 17, as the number of visits for 2 participants could not be retrieved), respectively.

*Adverse events.* Mild adverse events were reported by 5 participants and included bruises (*n* = 2), swelling (*n* = 1), blisters (*n* = 2), and pressure points (*n* = 1). For these individuals, a new scan was done and a new orthosis was printed. In one of these participants, the skin was too thin to prevent bruises resulting from the 3D-printed orthosis. Therefore, a new conventional leather orthosis was manufactured, and this person therefore dropped out during the follow-up.

## DISCUSSION

In this feasibility study in individuals with chronic hand conditions, newly made 3D-printed hand orthoses were similar to participants’ existing conventional hand orthoses with regard to ADL performance and quality of life. Satisfaction with the 3D-printed orthosis was higher than with the conventional orthosis, which is supported by the vast majority of those (79%) preferring the 3D-printed orthosis. Production time and costs of the 3D-printed hand orthoses were more than halved compared with conventional hand orthoses.

For the primary outcome, ADL performance, our results suggest that 3D-printed orthoses are similar to conventional orthoses. This is in line with findings from studies in individuals with osteoarthritis (10) and wrist pain (12), although these studies compared 3D-printed orthoses with plaster cast and prefabricated orthoses, respectively, which have different characteristics. Furthermore, it should be noted that these studies used other questionnaires (Quick Disabilities of Arm, Shoulder and Hand [Quick DASH] and the Upper Extremity Functional Status [UEFS] of the Orthotics and Prosthetics Users’ Survey) to assess ADL performance. While the UEFS has comparable ADL items to the DF-PROMIS-UE and MHQ-DLV ADL domain, the Quick DASH also contains items on hand function, social activities, and work, which could result in different findings. Although ADL performance with 3D-printed orthoses was somewhat reduced after 4 months’ follow-up as compared with conventional orthoses (mean difference DF-PROMIS-UE: 1.46; MHQ-DLV ADL domain: 4.99), the observed differences were far below the minimal important change (MIC) of both questionnaires used as reported in other studies (MIC DF-PROMIS-UE: from 3.6 to 6.3; MIC MHQ-DLV ADL domain: from 12 to 14.7) (26–30). One reason for the similarity in ADL performance in our study could be that the 3D-printed orthosis was made in accordance with the same type as the participant’s conventional orthosis. Thereby, functionality of the hand remained the same with both orthoses, which is also reflected in similar scores on the MHQ-DLV hand function domain. Since both ADL performance and hand functionality remained the same, this might also explain the similarity in the quality of life between the two orthoses.

While ADL performance and quality of life were practically similar between 3D-printed and conventional orthoses, orthosis satisfaction, as measured with the D-CSD, was significantly higher for the 3D-printed orthoses. This is in line with reported reasons for preferring the 3D-printed orthosis, which include comfort aspects such as a more precise fit, lower weight, and reduced transpiration. Higher satisfaction for 3D-printed orthoses was also demonstrated in a previous study in people with osteoarthritis that measured satisfaction with the D-QUEST including the services subscale in the total score (10), as well as in our previous case series by means of a self-designed, non-validated questionnaire (9). In our current study, we only considered the device subscale on the D-QUEST, which showed no significant difference in orthosis satisfaction between 3D-printed orthoses and conventional orthoses. This may reflect that the D-QUEST measures device satisfaction in general, whereas the D-CSD is specifically designed to measure orthosis satisfaction.

Production time and costs were more than halved for 3D-printed orthoses compared with conventional custom-fabricated orthoses, which is an important financial advantage of the 3D-printing technique. The production of 3D-printed orthoses eliminates several steps from the conventional manufacturing process (8), reducing labour time, which was also shown in our case series (9). Reduced labour time leads to lower costs of 3D-printed orthoses, as shown in this current study. Yet, it should be noted that only the production time of the first orthoses was analysed for both the 3D-printed and conventional hand orthoses. This could have led to an underestimation of labour time, and thus also an underestimation of the costs. It was shown that 5 individuals in our study had to have a 3D-printed orthosis re-manufactured, which may be due to the new technology of 3D-printing orthoses and the digital skills required by the orthotists. In clinical practice, however, conventional orthoses may also not fit properly immediately, requiring them to be remade. This was the case in one person in our sample. In a future study, labour time required to manufacture a new orthosis when the first orthosis does not fit properly should be incorporated for a more precise calculation of the costs. In addition, considering this was a feasibility study, we only assessed costs directly related to the manufacturing of the orthosis, but not other costs such as healthcare costs, informal costs, unpaid productivity costs, and costs related to productivity losses. For a complete overview of the efficiency of 3D-printing to manufacture hand orthoses, these costs should also be inventoried. Therefore, a cost-effectiveness study, including an economic evaluation, is warranted. When considering such a study, we suggest (*i*) a non-inferiority design with regard to the primary outcome ADL performance, (*ii*) using the custom short form of the DF-PROMIS-UE and D-CSD to assess ADL performance and orthosis satisfaction, respectively, as these questionnaires were the most responsive according to the reported effect sizes, and (*iii*) assessing outcomes at long-term follow-up (for example after 1 year), as orthoses are reimbursed every 2 years under the Dutch Healthcare Insurance Regulations.

### Strengths and limitations

This is the first study evaluating ADL performance and costs of 3D-printed hand orthoses compared with conventional custom-fabricated orthoses in chronic hand conditions. We included 2 assessments prior to the intervention and used 2 questionnaires to assess ADL performance, as well as to measure orthosis satisfaction. This provided information on possible anticipation effects between T1 and T2 and the effect sizes of the questionnaires, which can be used in determining assessment time points and using the most appropriate questionnaires in future studies. Furthermore, as this study was conducted in a heterogeneous sample of chronic hand orthotic users, wearing the 3 most commonly prescribed types of hand orthoses, it seems likely that the results can be generalized to the population of chronic hand orthotic users at large, although our results need to be confirmed in an adequately powered future (cost-)effectiveness study.

A limitation of our study is that the newly manufactured 3D-printed orthoses were compared with the custom-fabricated orthoses individuals were currently wearing. Selection bias may have occurred if, in particular, those who were dissatisfied with their conventional orthosis were willing to participate. Second, due to the fact that participants were dependent on their orthosis, no wash-out period was applied before starting the 3D intervention. Nonetheless, to minimize possible carry-over effects (31), follow-up measurements with the 3D-printed orthosis were not conducted directly after the delivery of the 3D-printed orthosis, but restricted to the latter part of the wearing period of the 3D-printed orthosis, that is, after 1 month and 4 months of using the orthosis. Finally, we did not assess limitations due to the presence of (new) comorbidities during the course of the study, e.g. reduced shoulder mobility, which could also affect ADL performance and quality of life, and might explain the heterogeneity of effects on an individual level. These limitations can be addressed in a future randomized clinical trial.

### Clinical implications

For the purpose of the study, the 3 parties (Spentys, the Department of Rehabilitation Medicine of Amsterdam UMC, and OIM Orthopedie) jointly developed standardized model scripts for the TO, WO, and WTO and established standardized operating procedures (SOPs) for the entire 3D manufacturing process. Future 3D-printed orthoses can be manufactured quickly and uniformly when these SOPs are used in clinical practice. Another advantage is that the scan of the hand and the design of the orthosis can be stored digitally, hence the orthosis can easily be reprinted. Yet, producing 3D-printed orthoses requires investment (e.g. scanner, software, and 3D printer) and orthotists need to be trained in specific digital skills. These factors could be barriers to the large-scale implementation of 3D-printing of hand orthoses in clinical practice (7).

In conclusion, this feasibility study in individuals with chronic hand conditions suggests that 3D-printed hand orthoses are similar to conventional custom-fabricated orthoses in terms of ADL performance, hand function, and quality of life. In addition, higher satisfaction related to improved comfort of use, and lower production time and costs were found in favour of the 3D-printed hand orthosis. An adequately powered randomized controlled cost-effectiveness study with long-term follow-up is warranted to confirm these findings.

## References

[1] Supan TJ. Chapter 4: Principles of fabrication. In: Hsu JD MJ, Fisk R., editor. AAOS atlas of orthoses and assistive devices. 4th ed. Philadelphia: Mosby Elsevier; 2008. p. 53–59.

[2] Jacobs M, Coverdale J. Concepts of Orthotic Fundamentals. In: Jacobs M, Austin N, editors. Orthotic intervention for the hand and upper extremity: splinting principles and process. Second ed. Baltimore, Philadelphia: Wolters Kluwer Health/Lippincott Williams & Wilkins; 2014. p. 2–25.

[3] Becker SJ, Bot AG, Curley SE, Jupiter JB, Ring D. A prospective randomized comparison of neoprene vs thermoplast hand-based thumb spica splinting for trapeziometacarpal arthrosis. Osteoarthritis Cartilage 2013; 21: 668–675. DOI: 10.1016/j.joca.2013.02.006 23458785

[4] Pizzi A, Carlucci G, Falsini C, Verdesca S, Grippo A. Application of a volar static splint in poststroke spasticity of the upper limb. Arch Phys Med Rehabil 2005; 86: 1855–1859. DOI: 10.1016/j.apmr.2005.03.032 16181954

[5] Videler A, Eijffinger E, Nollet F, Beelen A. A thumb opposition splint to improve manual dexterity and upper-limb functioning in Charcot-Marie-Tooth disease. J Rehabil Med 2012; 44: 249–253. DOI: 10.2340/16501977-0932 22366728

[6] Oud TAM, Lazzari E, Gijsbers HJH, Gobbo M, Nollet F, Brehm MA. Effectiveness of 3D-printed orthoses for traumatic and chronic hand conditions: a scoping review. PLoS One 2021; 16: e0260271. DOI: 10.1371/journal.pone.0260271 34793566 PMC8601455

[7] Schwartz DA, Schofield KA. Utilization of 3D printed orthoses for musculoskeletal conditions of the upper extremity: a systematic review. J Hand Ther 2023; 36: 166–178. DOI: 10.1016/j.jht.2021.10.005 34819255

[8] Barrios-Muriel J, Romero-Sanchez F, Alonso-Sanchez FJ, Rodriguez Salgado D. Advances in orthotic and prosthetic manufacturing: a technology review. Materials 2020; 13: 295. DOI: 10.3390/ma13020295 31936429 PMC7013385

[9] Oud T, Kerkum Y, de Groot P, Gijsbers H, Nollet F, Brehm MA. Production time and user satisfaction of 3-dimensional printed orthoses for chronic hand conditions compared with conventional orthoses: a prospective case series. J Rehabil Med Clin Commun 2021; 4: 1000048. DOI: 10.2340/20030711-1000048 33884150 PMC8054741

[10] Eyiis E, Mathijssen NMC, Kok P, Sluijter J, Kraan GA. Three-dimensional printed customized versus conventional plaster brace for trapeziometacarpal osteoarthritis: a randomized controlled crossover trial. J Hand Surg Eur 2023; 48: 412–418. DOI: 10.1177/17531934221146864 36650951

[11] Zheng Y, Liu G, Yu L, Wang Y, Fang Y, Shen Y, et al. Effects of a 3D-printed orthosis compared to a low-temperature thermoplastic plate orthosis on wrist flexor spasticity in chronic hemiparetic stroke patients: a randomized controlled trial. Clin Rehabil 2020; 34: 194–204. DOI: 10.1177/0269215519885174 31686529

[12] Kim SJ, Kim SJ, Cha YH, Lee KH, Kwon JY. Effect of personalized wrist orthosis for wrist pain with three-dimensional scanning and printing technique: a preliminary, randomized, controlled, open-label study. Prosthet Orthot Int 2018; 42: 636–643. DOI: 10.1177/0309364618785725 30009672

[13] GIPdatabank.nl. Aantal gebruikers 2017–2021, hulpmiddelencategorie C05: Orthesen [cited 12 December 2023]; Available from: https://www.gipdatabank.nl/databank?infotype=h&label=00-totaal&tabel=B_01-basis&geg=gebr&item=C05

[14] Bowen DJ, Kreuter M, Spring B, Cofta-Woerpel L, Linnan L, Weiner D, et al. How we design feasibility studies. Am J Prev Med 2009; 36: 452–457. DOI: 10.1016/j.amepre.2009.02.002 19362699 PMC2859314

[15] Eldridge SM, Chan CL, Campbell MJ, Bond CM, Hopewell S, Thabane L, et al. CONSORT 2010 statement: extension to randomised pilot and feasibility trials. Pilot Feasibility Stud 2016; 2: 64. DOI: 10.1186/s40814-016-0105-8 27965879 PMC5154046

[16] Oud T, Tuijtelaars J, Bogaards H, Nollet F, Brehm MA. Preliminary effectiveness of 3D-printed orthoses in chronic hand conditions: study protocol for a non-randomised interventional feasibility study. BMJ Open 2023; 13: e069424. DOI: 10.1136/bmjopen-2022-069424. PMC10083733 37024252

[17] Haan EA, Terwee CB, Van Wier MF, Willigenburg NW, Van Deurzen DFP, Pisters MF, et al. Translation, cross-cultural and construct validity of the Dutch-Flemish PROMIS® upper extremity item bank v2.0. Qual Life Res 2020; 29: 1123–1135. DOI: 10.1007/s11136-019-02388-2. 31894506

[18] Abma IL, Butje BJD, Ten Klooster PM, van der Wees PJ. Measurement properties of the Dutch-Flemish patient-reported outcomes measurement information system (PROMIS) physical function item bank and instruments: a systematic review. Health Qual Life Outcomes 2021; 19: 62. DOI: 10.1186/s12955-020-01647-y 33627157 PMC7905571

[19] Huijsmans R, Sluiter H, Aufdemkampe G. Michigan Hand Outcomes Questionnaire. FysioPraxis 2001: 38–41 (in Dutch).

[20] Chung KC, Pillsbury MS, Walters MR, Hayward RA. Reliability and validity testing of the Michigan Hand Outcomes Questionnaire. J Hand Surg Am 1998; 23: 575–587. DOI: 10.1016/S0363-5023(98)80042-7 9708370

[21] Arcidiacone S, Panuccio F, Tusoni F, Galeoto G. A systematic review of the measurement properties of the Michigan Hand Outcomes Questionnaire (MHQ). Hand Surg Rehabil 2022; 41: 542–551. DOI: 10.1016/j.hansur.2022.08.005 35995419

[22] Oud T, Tuijtelaars J, Schenk J, Nollet F, Brehm M-A. Validity and reliability of the Dutch translation of the OPUS’ client satisfaction with device module in chronic users of hand orthoses. Health and Quality of Life Outcomes 2023; 21: 93. DOI: 10.1186/s12955-023-02181-3 37605151 PMC10441692

[23] Demers L, Weiss-Lambrou R, Ska B. Development of the Quebec User Evaluation of Satisfaction with assistive Technology (QUEST). Assist Technol 1996; 8: 3–13. DOI: 10.1080/10400435.1996.10132268 10159726

[24] Demers L, Weiss-Lambrou R, Ska B. The Quebec User Evaluation of Satisfaction with Assistive Technology (QUEST 2.0): an overview and recent progress. Technology and Disability 2002; 14: 101–105. DOI: 10.3233/TAD-2002-14304

[25] Wessels RD, De Witte LP. Reliability and validity of the Dutch version of QUEST 2.0 with users of various types of assistive devices. Disabil Rehabil 2003; 25: 267–272. DOI: 10.1080/0963828021000031197 12623616

[26] Hoogendam L, Koopman JE, van Kooij YE, Feitz R, Hundepool CA, Zhou C, et al. What are the minimally important changes of four commonly used patient-reported outcome measures for 36 hand and wrist condition-treatment combinations? Clin Orthop Relat Res 2022; 480: 1152–1166. DOI: 10.1097/CORR.0000000000002094 34962496 PMC9263468

[27] Koopman JE, van Kooij YE, Selles RW, Slijper HP, Smit JM, van Nieuwenhoven CA, et al. Determining the minimally important change of the Michigan Hand outcomes Questionnaire in patients undergoing trigger finger release. J Hand Ther 2023; 36: 139–147. DOI: 10.1016/j.jht.2021.06.003 34312042

[28] Bernstein DN, Houck JR, Mahmood B, Hammert WC. Minimal clinically important differences for PROMIS physical function, upper extremity, and pain interference in carpal tunnel release using region- and condition-specific PROM Tools. J Hand Surg Am 2019; 44: 635–640. DOI: 10.1016/j.jhsa.2019.04.004 31126813

[29] Kazmers NH, Hung M, Bounsanga J, Voss MW, Howenstein A, Tyser AR. Minimal clinically important difference after carpal tunnel release using the PROMIS platform. J Hand Surg Am 2019; 44: 947–953 e941. DOI: 10.1016/j.jhsa.2019.03.006 31072663 PMC6829061

[30] Kazmers NH, Qiu Y, Ou Z, Presson AP, Tyser AR, Zhang Y. Minimal clinically important difference of the PROMIS upper-extremity computer adaptive test and QuickDASH for ligament reconstruction tendon interposition patients. J Hand Surg Am 2021; 46: 516–516 e517. DOI: 10.1016/j.jhsa.2020.11.007 33431194

[31] Sibbald B, Roberts C. Understanding controlled trials: crossover trials. BMJ 1998; 316: 1719. DOI: 10.1136/bmj.316.7146.1719 9614025 PMC1113275

